# Scientific Items

**Published:** 1881-11-20

**Authors:** 


### SCIENTIFIC ITEMS.

**To Hear the Grass Grow.**—At a recent meeting of the Silesian botanists, an apparatus made by Thomas and Lugel was exhibited which permits us to measure the rapidity of the growth of a plant. The latter is connected with an index which advances visibly and constantly, and exhibits the growth on a scale fifty times magnified. If this index be connected with an electric hammer, the current of which is interrupted as the index passes over the divisions of the circle, the growth of the plant will not only be visible, but also audible to the ear; hence the phrase "to hear the grass grow" will no longer be without a literal meaning.—*Jour. of Chem.*

**Cotton-Seed Oil.**—The American Consul at Naples reports that cotton-seed oil has already found its way into the remotest mountain villages of Italy, so that unadulterated olive oil is as rare there as here. If the resemblance is, as he says, so great that the most expert cannot detect the mixture, what real harm is done? Why not save the freight from here to Italy and back, as well as the double duties, give to it some less objectionable name than it now has, refine it most carefuly, and use it as salad oil? It is said that it is used in New Orleans for making artificial butter, and we could easily believe that the artificial butter would be better than the natural Southern butter.—*Ibid.*

**Giants and Dwarfs.**—(Revue d' Anthropologie.) The following is from the London Times of last year: "A committee of the London Anthropological Institute has been appointed to report on three persons who have been on exhibition at the Royal Aquarium, whose stature is very abnormal.

The first is a Chinese, named Chang, thirty-three years of age, who measures eight feet and two inches in height; sixty inches around the waist, and weighs 320 pounds. He is educated and speaks five languages. His type is Mongolian, and his features express good nature.

The second is a Norwegian, named Brustad, aged thirty-five years, whose stature reaches seven feet nine inches, his weight being 340 pounds.

The third, a dwarf, is named Chermach; he is forty-two years old, and is twenty-five inches in height. He speaks English fluently, gives a description of himself to his visitors and recites a Chinese elegy for them. He is the smallest dwarf ever seen."

In this the Times is in error, the smallest known is cited by Buffon from Birch, and measured forty-three centimeters (= 16¾ inches) at thirty-seven years of age. Another, Jeffery Hudgson, aged twenty years and measuring the same, is celebrated. He was presented by the Duchess of Buckingham to Queen Henriette Marie, of France. At the end of a dinner, he used to leap out of a pie, armed from head to foot, and sword in hand he would parade the table.

On the other hand, the greatest of the giants are the famous Finlander Caianu, who attained two meters, eighty-three c. m. (= 9 feet, 3 inches), an Austrian giant of two meters, 55 c. m. (= 8 feet, 4 inches), of whom a cast of the leg bone figured at the exposition of anthropology in 1878, and the Kalmuck, Ivan Louschkin, whose bones are in the Orfila Museum at Paris, who was two meters, 54 c. m. (= 8 feet, 3¾ inches) in height.—*Clinical Record.*

**Electricity of Rubber.**—An interesting illustration of the danger attending the manufacture of some kinds of rubber goods was shown in the origin of the fire which occurred in the Ætna Rubber Mills at Jamaica Plains. The cement which fastens the seams of rubber coats is largely made of naphtha. The mere act of lifting a piece of rubber cloth from a pile of half a dozen similar ones cut for garments, developed so much electricity that a spark was observed to escape. It came in contact with the naphtha cement or with gases arising from it, and instantly the whole room was in a blaze. Fortunately the fire was extinguished without destroying the mill, the loss being only about a thousand dollars.

It is not known that anything can be done to prevent the occurrence of another accident of precisely the same kind whenever all the atmospheric conditions are favorable. One would suppose, however, that a certain degree of dampness would remove all danger from that source.—*Weekly Journal.*

**Petroleum on Trees and Bushes.**—At a recent meeting of the California Academy of Sciences, Dr. H. Gibbons said that since he put petroleum on the trees in his garden they have grown better and faster than ever before, and given better roses than before. The petroleum seems to kill the scale insect. The handsomest rose he exhibited was from a bush which looked nearly dead a short time before. The petroleum was mixed with castor oil. It is applied sparingly, and great care taken that it does not run down the roots. Perhaps in a crude state the petroleum would be bad, even on the stalks; but mixed with the castor-oil it appears to be advantageous to the plant.—*Jour. of Chemistry.*

**Tincture of Chloride of Iron.**—Dr. Squibb, in Boston Journal of Chemistry, says: "In regard to the tincture of the chloride of iron, the last committee of revision of the U. S. P. made a mistake which is to be corrected in this revision. Tincture chloride of iron is not fit for use until at least six months old. I never send out any that is less than six months old, and have now changed to make it a year old. An important part of its therapeutic value depends upon ethers that are generated slowly from the large excess of HCl [hydrochloric acid] and the alcohol, and any one who will compare the sensible properties of an old with a recently made tincture will see how very different they are. The present U. S. P. therefore, in permitting the acid solution of the chloride to be kept and sold separately, so that the pharmacist can make up his tincture as he wants it, makes a great mistake, and on that account I have never made nor offered the solution of the U. S. P. for sale.

